# Is Encapsulated Medullary Thyroid Carcinoma Associated With a Better Prognosis? A Case Series and a Review of the Literature

**DOI:** 10.3389/fendo.2022.866572

**Published:** 2022-04-27

**Authors:** Andrea Contarino, Alessia Dolci, Marco Maggioni, Francesca Maria Porta, Gianluca Lopez, Uberta Verga, Francesca Marta Elli, Elisabetta Francesca Iofrida, Gianmaria Cantoni, Giovanna Mantovani, Maura Arosio

**Affiliations:** ^1^ Department of Clinical Sciences and Community Health, University of Milan, Milan, Italy; ^2^ Endocrinology Unit, Fondazione Istituto di Ricovero e Cura a Carattere Scientifico (IRCCS) Ca’ Granda Ospedale Maggiore Policlinico, Milan, Italy; ^3^ Pathology Unit, Fondazione Istituto di Ricovero e Cura a Carattere Scientifico (IRCCS) Ca’ Granda Ospedale Maggiore Policlinico, Milan, Italy; ^4^ Otolaryngology and Head and Neck Surgery Unit, Fondazione Istituto di Ricovero e Cura a Carattere Scientifico (IRCCS) Ca’ Granda Ospedale Maggiore Policlinico, Milan, Italy; ^5^ Endocrine Surgery Unit, Fondazione Istituto di Ricovero e Cura a Carattere Scientifico (IRCCS) Ca’ Granda Ospedale Maggiore Policlinico, Milan, Italy

**Keywords:** thyroid tumors, medullary thyroid carcinoma, tumor encapsulation, capsular invasion, calcitonin, desmoplastic stromal reaction

## Abstract

**Context:**

Medullary thyroid carcinoma (MTC) is a malignant neuroendocrine neoplasm that may spread to lymph nodes before the primary tumor is diagnosed; moreover, distant metastases are already present in about 10% of patients at diagnosis. Serum calcitonin (Ctn) usually reflects the spread of disease, thus orienting the extent of surgery and predicting the possibility of biochemical remission. Tumor size and vascular invasion are important prognostic factors, but little is known on the relationship between other histopathological features, such as the presence of a tumor capsule, and long term outcome of MTC.

**Purpose:**

To evaluate the prevalence of encapsulated tumors among MTCs and the association of tumor capsule with a favorable outcome after surgery.

**Methods:**

A retrospective observational single-center study was conducted together with a narrative review of the available literature.

**Results:**

Among 44 patients (27 female, 17 male; median age: 56 years) with MTC (6 hereditary, 37 sporadic) followed up at our center in the last four years (median follow-up: 29.2 months), seven (15.9%) showed an encapsulated tumor at histology and a clinical remission after surgery. None of them had nodal metastases and median preoperative Ctn (398 pg/mL, IQR 126.5–7336) did not differ significantly from that of the 14 patients (31.8%) with persistent disease after surgery (787 pg/mL, IQR 340.5–2905.5; p=0.633), although their tumor size was significantly higher (median 33 mm versus 16 mm respectively, p=0.036). Among patients with preoperative Ctn levels above 500 pg/mL (n=11), only two (18.2%) showed undetectable Ctn levels during follow-up, both having an encapsulated MTC (OR 0.000, p=0.02). Notably, they were two similar cases of large MTC (> 3 cm) with extensive hyalinization and calcification, associated with very high Ctn levels (> 13’500 and 1’100 pg/mL, respectively) but no nodal nor distant metastases, in complete remission after surgery although one of them carried the aggressive M918T somatic *RET* mutation.

**Conclusion:**

MTC rarely shows a tumor capsule, which seems to correlate with a better prognosis and absence of nodal metastases, regardless of *RET* or *RAS* mutational status. Among encapsulated MTCs (E-MTC), Ctn levels and tumor size are not predictive of persistence of disease after surgery.

## Introduction

Medullary thyroid carcinoma (MTC) is a rare neuroendocrine tumor originating from calcitonin-secreting thyroid C-cells (1). It accounts for 3-5% of all primary thyroid malignancies and occurs sporadically in 75-80% of cases. Activating germline mutations of the *RET* proto-oncogene are responsible for remaining hereditary forms, which include multiple endocrine neoplasia (MEN) syndromes type 2A and 2B (2). MTC shows variable clinical course but an overall more aggressive behavior, for different tumor cell lymphovascular dissemination compared to well-differentiated papillary and follicular thyroid carcinomas, and it is more prone to have lymph node and distant metastases at diagnosis (50-75% and 10%, respectively) (3).

Calcitonin (Ctn) serum concentration is a sensitive and specific biomarker useful for early detection of MTC (4). Furthermore, preoperative Ctn levels in MTC may be indicative of tumor burden, as with every increment of basal Ctn levels (above 20, 50 and 200 pg/mL, respectively) there is a successive involvement of the ipsilateral, contralateral paratracheal and bilateral laterocervical lymph node compartments, with upper mediastinal and distant metastases becoming more common above a basal Ctn threshold of 500 pg/mL (5). Therefore, Ctn is very useful for orienting the locoregional extent of surgery (after proper preoperative radiological staging) and predicting postoperative biochemical cure of patients with MTC (6). A similar relation with tumor size, number of lymph nodes metastases and outcome was seen for carcinoembryonic antigen (CEA) levels and, recently, also for procalcitonin (PCT) levels (5, 7).

Patients with intrathyroidal disease have a 10-year survival rate of 95.6%, whereas the presence of locoregional involvement or distant metastases at the time of diagnosis are associated with overall survival rates of 75.5% and 40%, respectively (8). Therefore, radical neck (thyroid and involved cervical lymph nodes) surgery represents the first-line therapy to achieve MTC cure (9). Systemic treatment is to be considered for those patients with progressive advanced disease (10). Current available drugs for MTC include multikinase inhibitors (MKIs) Vandetanib and Cabozantinib and new selective RET inhibitors Selpercatinib and Pralsetinib, but none of these have been shown to improve patients’ overall survival (OS) (11). Moreover, numerous side effects have frequently been reported and primary or acquired resistance mechanisms may be present (12).

Some histological features of the primary tumor have been proposed for predicting the outcome of MTC (13). Among these, lymphovascular invasion, intense desmoplastic stromal reaction (DSR), evidence of infiltrative tumor margins and extrathyroidal extension (ETE) (14, 15) significantly correlate with the presence of node metastases which is, in turn, the most relevant predictor of distant metastatic disease in MTC (16). On the contrary, the presence of a complete tumor capsule is a strong predictor of the absence of lymphatic spreading of the disease (17).

To date, just over a dozen published studies have described patients with encapsulated MTC (E-MTC) and correlated the presence and infiltration of the tumor capsule with the presence of lymph node metastases or disease remission after surgery, respectively. Very recently, Machens et al. (18) proposed the possibility of avoiding lymph node dissection in the case of well-encapsulated tumors without associated desmoplastic reaction, tested at intraoperative examination. Considering this interesting hypothesis, in the present article we have retrospectively researched cases of encapsulated tumors within the series of MTC patients in follow-up at our Institution to assess their staging at diagnosis and subsequent response to therapy. In addition, previously published research studies focusing on this histopathological issue have been reviewed and discussed.

## Material and Methods

A total of 53 consecutive patients with MTC underwent one or more follow-up visits at our Institution between January 2017 and January 2022. From this cohort, 44 patients with sufficiently detailed histopathological examination were selected, and samples of 26 MTCs diagnosed at Fondazione IRCCS Ca’ Granda Ospedale Maggiore Policlinico, Milan, Italy between 2010 and 2021 were identified and independently reviewed by three pathologists with experience of endocrine pathology, blinded to the patient lymph nodes status and clinical outcome. The study was conducted in accordance with the World Medical Association’s Declaration of Helsinki and approved by the local ethics committee.

All patients with a preoperative diagnosis of MTC underwent total thyroidectomy (except for one subject who was submitted to lobo-isthmectomy for surgical contraindications) and systematic central neck compartment lymphadenectomy. Patients with clinical evidence or suspicion of laterocervical metastases also underwent dissection of lateral neck compartments (ipsilateral or bilateral, as appropriate). Preoperative assessment of distant metastases was performed with total body CT or CT/PET in patients with Ctn levels above 500 pg/mL. The diagnosis of MTC was confirmed histologically and the following common pathological features were assessed: primary tumor size and extension (single focus or multifocal tumor), tumor margins, intratumoral gross calcifications, extrathyroid extension (ETE), vascular invasion, number of removed lymph nodes metastases, total removed lymph nodes. Tumoral encapsulation was defined as the presence of a fibrous rim of tissue enveloping the tumor and capsular invasion was defined as full-thickness tumor infiltration of the capsule into the adjacent thyroid tissue. DSR was defined as newly formed collagen-rich stroma into a peritumoral circumferential area of 0.5 cm from the tumor margins; it was evaluated semi-quantitatively by visually estimating the presence or absence of fibrosis. The TNM classification and tumor staging were performed according to the criteria described in the 8th Edition of American Joint Committee on Cancer (AJCC) TNM Classification of MTC (19).

The selected patients’ medical records were retrospectively assessed up until the last follow-up (January 2022). Pre- and postoperative serum levels of Ctn and CEA, when available, were determined using chemoluminescent (CLIA) and electrochemiluinescent (ECLIA) assays. Patients diagnosed at Fondazione IRCCS Ca’ Granda Ospedale Maggiore Policlinico, Milan, Italy between 2018 and 2021 also performed PCT dosage with ECLIA method (Elecsys BRAHMS PCT, normal values between 0.02 and 0.06 ng/mL). The follow-up was based on regular clinical examination, neck ultrasound imaging and serum Ctn and CEA measurement every 3 to 12 months, depending on the patient’s response to treatment. Further radiological investigations, such as neck-torax-abdomen CT and total body CT/PET were performed to assess any distant metastases in patients with permanently elevated or progressively increasing Ctn values ​​after surgery. Of the patients with persistent disease after the first surgery, three underwent a second surgery on the neck lymph nodes. At last control, patients were considered in remission when there was neither biochemical (basal Ctn levels < 2 pg/mL) nor structural evidence of disease.

Molecular genetics investigations to discover the presence of germline *RET* mutations on peripheral blood of all MTC patients were performed by targeted Sanger or NGS (in the last 5 years) sequencing. Somatic *RET* and *RAS* pathogenetic variants were tested on the genomic DNA extracted from FFPE samples of surgically resected E-MTCs. DNA was obtained with the MagMAX FFPE DNA/RNA Ultra Kit (Applied Biosystems, US) according to the manufacturer’s instructions and the analysis was performed by targeted NGS sequencing on the Illumina MiSeq platform, using the HaloPlex Target Enrichment System kit (Agilent Technologies, Santa Clara, CA) for the library preparation; data analysis, including alignment, categorization and annotation of variants, was done with the SureCall application (Agilent Technologies, Santa Clara, CA). Some extremely degraded FFPE-derived DNAs were pre-amplified with the SsoAdvanced PreAmp Supermix (BioRad, US) to obtain amplicons of sufficient quality for subsequent Sanger sequencing (BigDye Terminator v3.1, Applied Biosystems, US); target sequences were previously amplified with the high fidelity polymerase Takara Taq HS polymerase (Takara, Japan).

Statistical analysis was performed with GraphPad Prism (version 9.3.1). Quantitative variables were expressed as medians with interquartile ranges (IQR) and complete ranges (from minimum to maximum) and were compared with the two-tailed Mann-Whitney U test. Qualitative variables were presented as absolute and relative (percentage) frequencies and were tested with a Chi-square test or Fisher’s exact test. Odds ratios (OR) were expressed together with their 95% confidence interval (95%CI). Correlations between quantitative variables were assessed by calculating Pearson’s correlation coefficient. The level of statistical significance (two-tailed) was set at p < 0.05.

## Results

Between January 2017 and January 2022, a total of 53 patients with MTC underwent a follow-up visit at our Institution. Among them, we retrieved complete pre- and postoperative medical records for 44 patients (female to male ratio: 27/17) who were diagnosed with MTC between February 1996 and August 2021 (median age at thyroidectomy of 56 years, IQR 46.5–66 years).

As shown in **Table 1**, preoperative serum Ctn was available for 37 of 44 patients of the study cohort and its median level was 184 pg/mL (IQR 75 – 720.5 pg/mL). Ctn was significantly correlated with tumor size (r = 0.723, 95%CI 0.521–0.848, p < 0.001) but not with the number of positive lymph nodes (r = 0.272, p = 0.109). DNA analysis for germline *RET* mutations showed 4 cases of hereditary MTC in the context of a MEN2A syndrome and 2 cases of FMTC (belonging to four different families).

**Table 1:** Clinical and histopathological characteristics of the study cohort.

No. of patients	44
Age at thyroidectomy, years [median, (IQR), range]	56 [46.5–66] (7–78)
Gender, no. of female/male patients	27/17
Hereditary/sporadic MTC	6/38
Preoperative Ctn level, pg/mL [median, (IQR), range]*	184 [75–720.5] (12.6–13540)
Primary tumor size, mm [median, (IQR), range]	14 [8–21] (1–65)
Single focus/multifocal tumor	38/6
No. of patients with intratumoral gross calcifications (%)	6 (13.6)
No. of patients with desmoplastic stromal reaction, DSR (%)**	18/26 (69.2)
No. of patients with extrathyroid extension, ETE (%)	4 (9.1)
No. of patients with vascular invasion (%)	10 (22.7)
No. of patients with nodal disease, N+ (%)	15 (34.1)
N+ in the central compartment, N1a	15 (100)
N+ in the laterocervical compartment, N1b	9 (60)
No. of node metastases removed, total [median, (IQR), range]***	147 (0) [0–2] (0–39)
No. of nodes removed, total [median, (IQR), range]***	544 (7) [1–22] (0–82)
AJCC TNM 8th edition clinical staging (%)	
Stage I	22 (50)
Stage II	7 (15.9)
Stage III	6 (13.6)
Stage IVA	9 (20.5)
Stage IVB	0 (0)
Stage IVC	0 (0)
No. of patients with biochemical cure (%)	30 (68.2)
Follow-up, months [median, (IQR), range]	29.2 [15.9–80.7] (0.3–350.6)

AJCC TNM, American Joint Committee on Cancer Tumor-Node-Metastases; Ctn, calcitonin; IQR, interquartile range.

*Preoperative Ctn levels were available for 37 of 44 patients of the study cohort.

**DSR was evaluated on the 26 samples of MTCs diagnosed at Fondazione IRCCS Ca’ Granda Ospedale Maggiore Policlinico, Milan, Italy.

***Number of removed and metastatic node was available for 43 of 44 patients of the study cohort.

**Figure 1:**
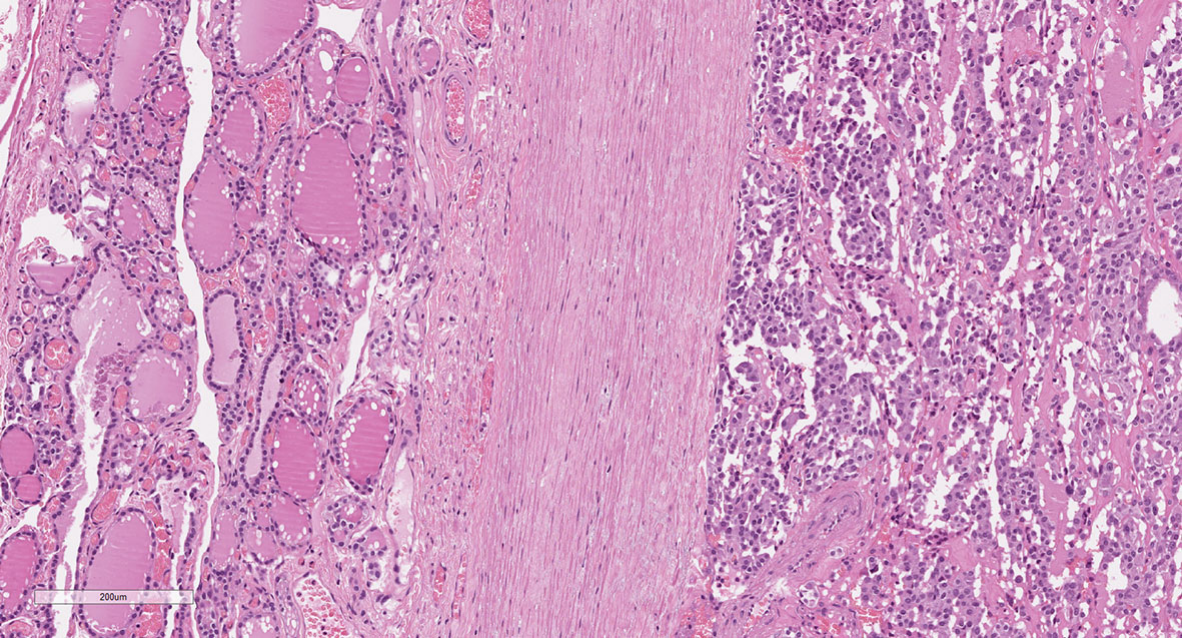
Medullary thyroid carcinoma showing a tumor capsule. The neoplastic cells (right) are demarcated from the normal thyroid parenchima (left) by a fibrous capsule (center). (Hematoxylin and eosin, original magnification 10x).

After histopathological examination, median primary tumor size was 14 mm (IQR 8–21 mm) and multifocality was present in 6 (13.6%) cases, five of whom showed bilateral tumor foci. We found that 7 of 44 MTCs had a tumor capsule (**Figure 1**). Among the non-encapsulated (NE-MTC) tumors (84.1%), defined by the total absence of a capsule surrounding the tumor, infiltrative margins were reported in 14 and expansive or well-defined margins in 14 out of 28 cases. Concerning other histopathological findings, 6 out of 44 (13.6%) exhibited diffuse (50%) or focal (50%) intratumoral gross calcifications, vascular invasion was observed in 10 (22.7%) and ETE in 4 (9.1%) of 44 cases. Fifteen (34.1%) patients had histologically confirmed lymph node metastases (pN1) at initial surgery, in all cases involving the central compartment (VI level) of the neck and in 60% of cases also the laterocervical compartment. Peritumoral desmoplasia was present in 18 of the 26 (69.2%) reviewed MTC specimen at our Institution. Half of these were associated with lymph node metastases (positive predictive value of 50%), whereas no DSR-negative cases had lymph node metastases (negative predictive value of 100%, p = 0.023) neither at primary surgery nor during the follow-up. According to the 8^th^ edition AJCC TNM Staging System, 22 (50%) patients had a Stage I tumor after surgery, 7 (15.9%) patients were at Stage II, 6 (13.6%) at Stage III and 9 (20.5%) at stage IVA. At diagnosis, as well as at the last outpatient visit, no radiologically proven distant metastases were detected.

At the last follow-up (median follow-up period of 29.2 months, IQR 15.9–80.7), 30 (68.2%) patients (including 3 of the 15 pN1 patients) achieved biochemical cure, 13 (29.5%) showed a biochemical incomplete response and a single patient (2.3%) had a structural incomplete response with stable disease. Statistically significant predictive factors of persistence of disease after primary surgery were vascular invasion (OR infinity, 95%CI 12.3–infinity, p < 0.0001), lymph nodes involvement (OR 54, 95%CI 6.99–279.2, p < 0.0001) and ETE (OR infinity, 95%CI 2.29–infinity, p = 0.007).

### Non-Encapsulated MTCs

Clinical and pathological features of NE-MTC patients (84.1%) were reported in the right side of **Table 2**, subgrouping them according to the presence or absence of biochemical remission at last visit (23 ‘cured’ and 14 ‘non-cured’ patients).

**Table 2:** Clinical and histopathological characteristics of encapsulated (all cured) and non-encapsulated MTC (cured and not cured).

Tumor capsule	Present	Absent	
No. of patients (%)	7 (15.9)	37 (84.1)	
**Biochemical cure**	**Cured (n = 30, 68.2%)**		**Not cured (n = 14, 31.8%)**
No. of patients (%)	7 (100)	23 (62.2)	14 (31.8)
Age at thyroidectomy, years [median, (IQR), range]	57 [34–60] (27–65)	56 [48–69] (7–78)	55 [45.3–66] (31–77)
Gender, no. of female/male patients	3/4	19/4	5/9
Hereditary/sporadic MTC	0/7	2/21	4/10
Preoperative Ctn level, pg/mL [median, (IQR), range]	398 [126.5–7336] (102–13540)	75 [42.2–127] (12.6–337)	787 [340.5–2905.5] (90.2–9916)
Primary tumor size, mm [median, (IQR), range]	33 [20–38] (14–65)	8.6 [6–15] (1–35)	16 [12–22.3] (7–41)
No. of patients with multifocal MTC (%)	0 (0)	2 (8.7)	4 (28.6)
No. of patients with intratumoral gross calcifications (%)	3 (42.9)	0 (0)	3 (21.4)
No. of patients with desmoplastic stromal reaction, DSR (%)	2/6 (33.3)	7/11 (63.6)	9/9 (100)
No. of patients with extrathyroid extension, ETE (%)	0 (0)	0 (0)	4 (28.6)
No. of patients with vascular invasion (%)	0 (0)	0 (0)	10 (71.4)
No. of patients with nodal disease, N+ (%)	0 (0)	3 (13)	12 (85.7)
N+ in the central compartment, N1a	0 (0)	3 (100)	12 (100)
N+ in the laterocervical compartment, N1b	0 (0)	0 (0)	9 (60)
No. of node metastases removed, total [median, (IQR), range]	0 (0)	7 (0) [0] (0–3)	140 (7) [2–15.5] (0–39)
No. of nodes removed, total [median, (IQR), range]	72 (7) [1–13] (0–39)	92 (1) [0–7] (0–22)	380 (31) [15.5–48] (6–82)
AJCC TNM 8th edition clinical staging (%)			
Stage I	2 (28.6)	18 (78.3)	2 (14.3)
Stage II	5 (71.4)	2 (8.7)	0 (0)
Stage III	0 (0)	3 (13)	3 (21.4)
Stage IVA	0 (0)	0 (0)	9 (64.3)
Stage IVB	0 (0)	0 (0)	0 (0)
Stage IVC	0 (0)	0 (0)	0 (0)
Follow-up, months [median, (IQR), range]	18.9 [16.1–23.9] (11–132.8)	54.8 [19.6–112] (3.5–350.6)	20.9 [6.3–47.1] (0.3–158.3)

AJCC TNM, American Joint Committee on Cancer Tumor-Node-Metastases; Ctn, calcitonin; IQR, interquartile range.

There were no significant differences in term of germline *RET* mutations (p = 0.174) and patients’ age when comparing patients in remission and those not in remission, whereas a statistically significant difference was noted concerning the preponderance of males among those non-cured (64.3% versus 17.4% among cured patients, p = 0.006).

Higher preoperative serum Ctn (p < 0.0001), greater tumor size (p = 0.008) and presence of ETE (p = 0.015) were significantly associated with lack of biochemical cure after surgery. Multifocality was not different between the two groups of NE-MTC patients. Vascular invasion was observed only in non-cured MTCs (p < 0.0001) and lymph nodes involvement was significantly more frequent in this subgroup of patients (12/15 positive nodes in non-cured versus 3/25 in cured patients, p < 0.0001). DSR was detected in 9 out of 9 (100%) non-cured NE-MTCs, almost always associated with lymph node metastases (88.9%), and in 63.6% (7/11) of cured NE-MTCs, only in one case (14.3%) associated with lymph node metastases.

Among all NE-MTCs, a significant correlation was found between serum Ctn at diagnosis and the number of node metastases removed (r = 0.515, 95%CI 0.197–0.735, p = 0.003).

### Encapsulated MTCs

Prevalence of encapsulated tumors in the present cohort of patients with histologically proven MTC was 15.9% (95%CI 5.1– 26.7%). Full-thickness invasion of the capsule was detected in 3 (42.9%) cases but such invasion was observed only in a single focus per case. No E-MTC was associated with nodal or distant metastases and all seven patients achieved biochemical and structural remission after neck surgery (excellent response).

All E-MTCs were sporadic tumors and concerned male patients in 57.1% and female in 42.9%, with a median age at thyroidectomy comparable with that of the entire study cohort (57 years, IQR 34–60 years).

**Figure 2:**
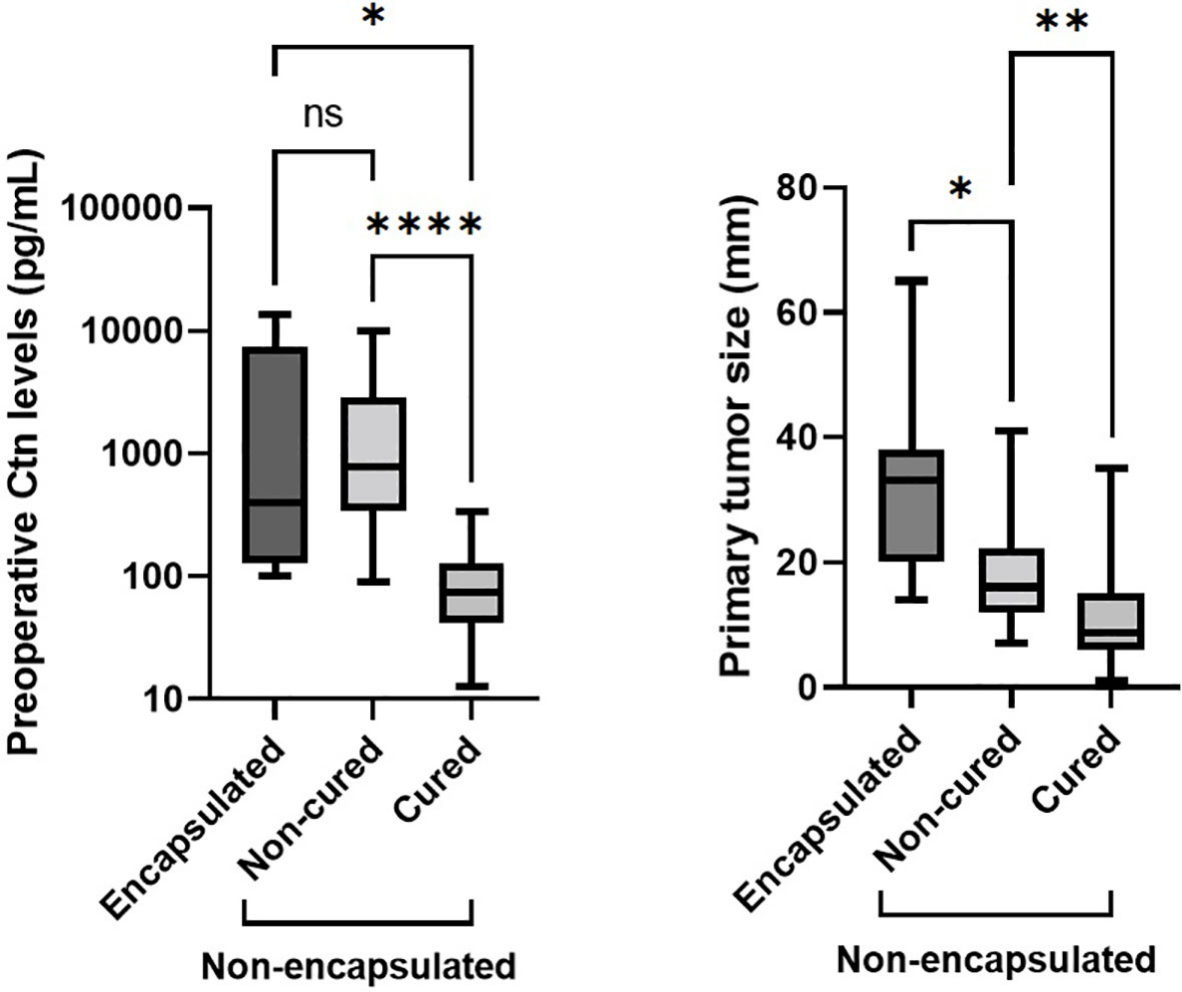
Comparing E-MTCs with non-cured NE-MTCs (**Table 2**), there were no significant differences in term of preoperative Ctn levels (p = 0.633, left side) but there were for primary tumor size (p = 0.036, right side), which was higher in the former (median 33 mm versus median 16 mm, respectively). Among NE-MTCs, higher preoperative serum Ctn (p < 0.0001) and greater tumor size (p = 0.008) were seen in non-cured patients than those cured. Ctn, calcitonin; ns, not significant. The asterisks refer to the different p values indicated in the caption of the figure (*p = 0.036, **p = 0.008, ****p < 0.0001).

Comparing E-MTCs with non-cured NE-MTCs (**Table 2**), there were no significant differences in term of preoperative Ctn levels (median 398 pg/mL, IQR 126.5–7336 pg/mL versus median 787 pg/mL, IQR 340.5–2905.5 pg/mL respectively, p = 0.633) but there were for primary tumor size (median 33 mm, IQR 20–38 mm versus median 16 mm, IQR 12–22.3 mm respectively, p = 0.036), as shown in **Figure 2**. However, E-MTCs did not show multifocality, extrathyroidal extension nor vascular invasion (p = 0.004). One case had a large central cystic component, whereas intratumoral gross calcifications were detected in 3 out of 7 E-MTCs (42.9%). DSR was significantly more present among the NE-MTCs examined (76.9%) compared to the E-MTCs (23.1%, p = 0.029). Among the latter, the two cases associated with peritumoral desmoplasia (only mild grade) both showed focal capsular invasion. Unlike non-cured NE-MTCs, in which AJCC/TNM stages III and IV prevailed (p = 0.003), E-MTCs were only stage I (28.6%) and Stage II (71.4%) tumors, depending exclusively on their size.

Tumor tissue mutational status was known for four (57.1%) of the E-MTCs: one case was positive for the two *RET* polymorphisms Gly691Ser (rs1799939) and Arg982Cys (rs17158558), other two cases showed the previous two *RET* variants combined with the somatic Met918Thr mutation (p.G691S/M918T/R982C compound genotype), whereas the last one was both *RET* and *RAS* wild-type. The remaining samples were not analyzable despite appropriate DNA amplification techniques.

### MTCs With Preoperative Ctn > 500 pg/mL

Eleven of 37 (29.7%) MTCs of the present study were associated to preoperative serum Ctn levels higher than 500 pg/mL (median 1730 pg/mL, IQR 787–3145 pg/mL), with a median CEA of 44.2 ng/mL (IQR 29–120 ng/mL) and a median PCT of 45.9 ng/mL (IQR 10.9–65.9 ng/mL). Their clinical and histopathological characteristics as well as response to surgery were reported in **Table 3**.

**Table 3:** Clinical and histopathological characteristics and response to surgery of MTCs presenting with preoperative serum Ctn levels above 500 pg/mL.

ID	Sex	Age (yrs)	Preoperative markers		Tumor size (mm)	Tumor capsule	ETE	Vascular invasion	Lymph nodes involvement	AJCC TNM 8th ed. staging	Clinical setting	Postoperative markers		Cured
			Ctn (pg/mL)	CEA (ng/mL)								Ctn (pg/mL)	CEA (ng/mL)	
#29	F	77	535	52.4	11	No	No	No	No	pT1b, N0, M0	sMTC	8.7	2.5	No
#23	F	77	654	*N/A*	20	No	No	Yes	No	pT1b, N0, M0	sMTC	348	34.3	No
#24	M	55	787	41.1	35	No	Yes	Yes	Yes	pT4a(m), N1b, M0	sMTC	92.6	16.3	No
#30	M	31	996	18.1	8	No	No	Yes	Yes	pT1a(m), N1b, M0	sMTC	320	6.9	No
#21	M	59	1132	88.0	33	Yes	No	No	No	pT2, N0, M0	sMTC	< 2	3.8	Yes
#10	M	66	1730	39.9	14	No	Yes	Yes	Yes	pT1b, N1b, M0	sMTC	88.3	1.8	No
#20	F	49	2815	44.2	41	No	No	Yes	Yes	pT3a, N1b, M0	FMTC	375	5.0	No
#4	M	55	2996	13.0	19	No	No	Yes	Yes	pT1b, N1b, M0	MEN2A	485	4.2	No
#39	F	61	3145	*N/A*	26	No	No	No	Yes	pT2, N1a, M0	sMTC	16.1	2.7	No
#3	M	46	9916	339	21	No	No	Yes	Yes	pT2, N1b, M0	sMTC	184	7.5	No
#40	M	60	13540	152	65	Yes	No	No	No	pT3a, N0, M0	sMTC	< 2	4.3	Yes

AJCC TNM, American Joint Committee on Cancer Tumor-Node-Metastases; CEA, carcinoembrion antigen; Ctn, calcitonin; ETE, extrathyroid extension; FMTC, familial medullary thyroid carcinoma; MEN2A, multiple endocrine neoplasia type 2A; N/A not assessed; sMTC, sporadic medullary thyroid carcinoma; Yrs, years.

Median primary tumor size was 21 mm (IQR 14–35 mm), vascular invasion and lymph node metastases were observed in 7 (63.6%) and ETE in 2 (18.2%) of 11 cases. Biochemical cure was reported only in two of the high preoperative Ctn-associated MTCs and both were encapsulated, so the presence of a tumor capsule was predictive of disease remission in this specific subgroup (OR 0.000, 95%CI 0–0.3, p = 0.02) but not in the entire MTC cohort (OR 0.000, 95%CI 0–1.185, p = 0.078). Notably, both E-MTCs were largely replaced by sclero-hyaline tissue with abundant intratumoral calcifications at the histological examination.

## Discussion

Tumoral encapsulation is a well-known important, if not fundamental, element for the pathological definition of follicular cell-derived thyroid tumors. According to the 4^th^ edition WHO Classification of Tumors of Endocrine Organs, published in 2017 (20), the detection of a whole and preserved tumor capsule in thyroid lesions allows to define benign tumors such as follicular adenoma and new borderline tumors such as the “follicular tumor of uncertain malignant potential” (FT-UMP) and the “non-invasive follicular thyroid neoplasm with papillary-like nuclear features” (NIFTP), which share a more indolent behavior and a favorable prognosis (21). Among malignant tumors, the presence of an encapsulated nodule, without evidence of the invasion of the tumor capsule, seems to be an independent prognostic factor for a good prognosis also in the classical variant of papillary thyroid carcinomas (encapsulated non-invasive CV-PTC), according to recent studies (22). Furthermore, it is known that follicular thyroid carcinomas (FTC) showing limited capsular infiltration, but not foci of vascular invasion (so called minimally invasive FTC), have a low recurrence risk after surgery (23).

Against so many evidences regarding both the diagnostic and prognostic value of tumoral encapsulation in differentiated thyroid tumors of follicular origin, only a limited number of studies (including several not recent case reports) have investigated the possible correlation between the presence of a complete tumor capsule with the clinical behavior and outcome in the setting of MTC. As shown in **Table 4**, so far fifteen studies on the pathological features of MTCs have described the presence of about 30% grossly and/or microscopically encapsulated tumors, of which at least half surrounded by a continuous and/or invasion-free capsule. Five (2.9%) cases were reported to have an extra-thyroidal involvement, exclusively limited to neck lymph nodes, but it was not known whether tumor capsule was intact or infiltrated or neither. Only ten (66.7%) of the 15 selected studies also analyzed clinical data on the follow-up and treatment response of patients with E-MTC. Where available, these studies showed biochemical remission of disease in almost all cases (98.9%).

**Table 4:** Review of previously published reports and series of E-MTCs.

Author, year [reference]	Description of reported E-MTCs (with definition of encapsulation when available)	E-MTC (%)	Capsule integrity (%)	Tumor size (mm)	Metastatic MTC (%), site	Biochemical Cure (%)
Williams et al., 1966 (24)	MTC sharply demarcated from the surrounding thyroid tissue by a complete or partial fibrous capsule	9/67 (13.4)	2/9 (22.2)	*NR*	*NR*	*NR*
Beskid, 1979 (25)	“C cell adenoma” surrounded by a thick hyaline capsule with no infiltration of the capsule by the nodule cells	1/1 (100)	1/1 (100)	40	0	1/1 (100)
Kodama et al., 1988 (26)	“C cell adenomas” (one with incomplete very thin capsule without infiltrative growth) with positive Ctn and no CEA staining; low serum CEA	1/2 (50)	0/1 (0)	40 (both)	0	2/2 (100)
Driman et al., 1991 (27)	MTC surrounded by a distinct fibrous capsule with weak Ctn, CEA and CgA staining; normal-high serum Ctn and low serum CEA	1/1 (100)	*NR*	20	0	1/1 (100)
Ozkara et al., 2002 (28)	Encapsulated (intact capsule) papillary variant MTC with extensive cystic degeneration and positive Ctn, CEA, CgA staining	1/1 (100)	1/1 (100)	40	0	1/1 (100)
Miccoli et al., 2007 (17)	MTCs divided in completely encapsulated and non-encapsulated	18/70 (34.6)	18/18 (100)	*NR*	0	18/18 (100)
Santosh et al., 2011 (29)	Well-encapsulated HTA-like variant MTC with positive Ctn staining	1/1 (100)	1/1 (100)	30	0	1/1 (100)
Bhat and Jena, 2012 (30)	Well-encapsulated HTA-like variant MTC with positive Ctn staining	1/1 (100)	1/1 (100)	30	0	*NR*
Aubert et al., 2018 (14)	MTCs with or without tumor capsule (*not otherwise specified*)	21/54 (38.9)	*NR*	*NR*	3 (14.3), N	18/18 (100)
Cipri et al., 2019 (31)	Encapsulated microMTC with positive Ctn and CgA staining; undetectable serum Ctn	1/1 (100)	*NR*	≤ 10	0	1/1 (100)
Censi et al., 2019 (32)	Well-encapsulated “borderline tumor” (between adenoma and carcinoma) with weak CEA and no CgA staining; low serum CEA	1/1 (100)	1/1 (100)	70	0	1/1 (100)
Alzumaili et al., 2020 (13)	MTCs divided in completely encapsulated/well circumscribed, partially encapsulated or totally lacking a capsule; capsular invasion defined as complete tumoral penetration of the capsule	26/143 (18.2)	8/26 (30.8)	*NR*	*NR*	NR*
Singh et al., 2020 (33)	Encapsulated papillary variant MTC with extensive cystic degeneration and positive Ctn and CgA staining	1/1 (100)	*NR*	80	1 (100), N	*NR* ^§§^
Moura et al., 2021 (15)	Collateral reporting of encapsulated MTC (*not otherwise specified*)	8/65 (12.3)	*NR*	*NR*	1 (12.5), N	*NR*
Machens et al., 2021 (18)	Tumor capsule integrity was classed into 5 subgroups: tumor capsule evenly demarcated; tumor capsule irregular but intact, with or without invasion; breach of the tumor capsule with ≤3 tumor extensions measuring ≤3 mm in width; breach of the tumor capsule with >3 tumor extensions or one tumor extension measuring >3 mm in width; diffuse tumor growth without tumor capsule.	81/139 (58.3)	47^§^/81 (58)	Between 7 and 12^§§^ (median)	0	45/46^§§§^ (97.8)
Sum of published cases		172/548 (31.4)	80/140 (57.1)	–	5 (2.9), N	89/90 (98.9)
Present series		7/44 (15.9)	4**/7 (57.1)		0	7/7 (100)
Total cases		179/592 (30.2)	84/147 (57.1)	–	5 (2.8), N	96/97 (98.9)

E-MTC, encapsulated medullary thyroid carcinoma; CEA, carcinoembrion antigen; CgA, cromogranin A; Ctn, calcitonin; HTA, hyalinizing trabecular adenoma; N, nodal metastases; NR, not reported.

*Tumor encapsulation improved loco-regional free survival but had no effect on disease specific survival and distant metastasis free survival.

^§^ Included cases with tumor capsule evenly demarcated, irregular but intact, or with less than 3 tumor extensions < 3 mm in width.

^§§^Only tumors with size ≤ 25 mm on histopathologic evaluation were included in the entire study.

**Only encapsulated tumors without full-thickness capsular invasion (albeit focal).

^§§§^One patient lost at follow-up.

According to their apparent more benign prognosis (than typical MTC), these encapsulated thyroid lesions have been named “C-cell adenomas” by some Authors (25, 26, 32) for the lack of malignant morphological features commonly found in MTC (mainly infiltrative growth pattern). However, they did not better define a reproducible pathological or molecular profile typical of this entity. A possible shared element between these cases could be the association with low serum CEA levels. Although less sensitive and specific than Ctn, CEA levels tend to increase with the disease stage in MTC. However, MTC associated with normal CEA values have been described both at diagnosis (some of which with ascertained lymph node metastases) and at the time of relapse (34), so that negativity to CEA can hardly be considered as a marker of benign behavior.

These promising albeit limited data have led some Authors (Miccoli et al.) to hypothesize the possibility of reconsider the extension of MTC surgical treatment on the basis of the lack of a preserved tumor capsule, that may be intraoperatively revealed by a frozen section analysis (17).

Other morphological parameters have been proposed as useful intraoperative markers to exclude node involvement and thereby to modulate the extent of the surgery in MTC patients, such as DSR (35). Peritumoral desmoplasia is defined as the presence of a newly formed fibrotic stroma surrounding the invasive epithelial tumor cells and can be demonstrated in the majority of MTCs (approximately 80%). In the remaining 20% of sporadic MTCs, as well as in a number of hereditary MTCs, DSR is completely lacking; these cases, which are usually well circumscribed but not necessarily enveloped by a tumor capsule, are typically associated with a very low metastatic potential (36). Combining both the favorable features of tumor encapsulation and absence of peritumoral desmoplasia, very recently Machens et al. proved that patients with E-MTC without associated DSR (or minimal/low desmoplasia), if confirmed on frozen section analysis by experienced pathologists, could avoid even routine central compartment lymph node dissection (18).

In the present study, we have retrospectively researched cases of encapsulated tumors within a series of MTC patients in follow-up at our Institution to assess their staging at diagnosis and subsequent response to therapy. To this purpose, we studied 44 cases of MTC, both hereditary (13.6%) and sporadic (86.4%) cases, and found that prevalence of E-MTC was 15.9%.

Within the entire cohort, preoperative Ctn significantly correlated with tumor size but not with the number of positive lymph nodes; excluding encapsulated tumors from these, however, a significant correlation was found between serum Ctn levels and the number of node metastases. This is because, although Ctn levels of E-MTCs were not significantly different from those of non-cured NE-MTCs (**Table 2**), none of the E-MTCs was associated with nodal or distant metastases, so they were only Stage I-II tumors. Furthermore, they did not show ETE nor vascular invasion, both histological features known to be associated with lymph node involvement in MTC, and all affected patients achieved clinical remission after the first surgical treatment. In the present study (**Table 2**), vascular invasion, lymph nodes involvement and ETE were statistically significant predictive factors of persistence of disease after primary surgery.

Peritumoral desmoplasia was a frequent finding among the reviewed MTC of the present study, always but not exclusively detected in non-cured NE-MTC, where it was almost invariably associated with lymph node metastases. DSR-negative MTCs did not involve lymph nodes neither at primary surgery nor during the follow-up, confirming the negative predictive value already described by various Authors. In support of this evidence, in a large retrospective study of 360 patients with MTC who underwent intraoperative frozen-section analysis before surgery, patients with DSR-negative tumor (18%) did not undergo lateral lymph nodes dissection and all maintained biochemical remission for up to 100 months after surgery. Patients with an intraoperative diagnosis of a DSR in the MTC specimen (82%) underwent total thyroidectomy and bilateral central and functional lateral neck dissection; in this group, lymph node and distant metastases were present in 31% and 6% of patients, respectively. As no patient in the DSR-negative group presented with LN metastases in any compartment (negative predictive value of 100%) and each of them had an excellent long-term prognosis, Authors proposed to avoid lateral neck surgery in MTC patients with intraoperative frozen-section negative for DSR (37). Among the E-MTCs of the present study, only two cases showed a mild grade DSR, and both were associated with focal capsular invasion.

Among NE-MTCs, male gender was significantly associated with lymph nodes metastases (but not with larger primary tumor size) and with persistence of disease after surgery. According to the current literature, men with MTC present with larger tumors and are less likely to have localized disease (38), since male sex was recognized as a possible risk factor for lateral neck lymph node metastasis (39). Male gender independently predicts worse overall survival in MTC, even if both disease burden at initial surgery and biochemical response to surgery appear to be stronger prognostic factors (40). Gender differences in term of MTC presentation and outcome could be attributed to a later diagnosis in men, for behavioral reasons and possibly for the lower tendency to perform thyroid tests compared to women, although an underlying biological explanation has been proposed recently but not yet confirmed in larger studies (41). Notably, men and women were almost numerically equal among E-MTC patients in the present study.

Primary tumor size was higher for E-MTCs than non-cured NE-MTCs, so neither serum Ctn nor tumor diameter were predictive of persistence of disease when tumor encapsulation was present (**Figure 2**). It was hypothesized that tumor lymphatic dissemination might be correlated with an infiltrative behavior, depending on the ability of the tumor cells to invade lymphatic vessels located in surrounding normal thyroid tissue (42). Conversely, encapsulated tumors are prevented from loco-regional dissemination due to the containing effect exerted by the capsule itself, and tend to reach much larger size as a result of their expansive growth pattern.

The different predictive value of preoperative Ctn between E- and NE-MTC is particularly evident in the patients subgroup with serum Ctn levels higher than 500 pg/mL (**Table 3**). Above this threshold, distant metastases become very common while biochemical cure rate is progressively reduced (6). No distant metastases were detected in the present series and tumor encapsulation was a statistically significant predictive factor of biochemical remission in this specific subgroup, but not in the entire MTC cohort. Among high preoperative Ctn-associated MTCs, only the two E-MTCs showed undetectable postoperative Ctn levels during a mean follow-up period of 17.4 months (versus 20.1 months in MTCs with persistent disease). It is noteworthy that they were two similar cases of large MTC characterized by extensive tumoral hyalinization and calcification, associated with very high preoperative Ctn and CEA levels, in complete remission after surgery although one of them carried the aggressive M918T somatic *RET* mutation.

Somatic *RET* mutations are present in 40-50% of sporadic MTC (sMTC), with the most common occurring in codon M918 (which is present in up to 90% of *RET*-positive cases) and in codon C634 (43). Recently, activating point mutations in *RAS* genes (H-, K-, and NRAS) have been described predominantly in *RET*-negative sMTC, with a percentage ranging from 10 to 60% depending on the different series (44) but a better prognosis than those harboring *RET* mutations or presenting no mutations. Trying to define a genotype–phenotype correlation in MTC, tumors with somatic p.Met918Thr *RET* mutation and those having no detectable *RET* or *RAS* mutations have been typically associated with lymphovascular invasion, extrathyroidal extension and more advanced stages of disease (15). It is not known whether this more aggressive behavior is maintained even in the presence of more favorable pathological features, such as the tumor capsule. So far only two studies (15, 32), in addition to the present one, have analyzed the mutational status of sporadic E-MTC (**Table 5**). Overall, four cases (30.8%) carried pathogenic mutations in *RET* exons 10 and 11 (C620S and C630S) and three (23.1%) the aggressive somatic M918T mutation (isolated or in combination with other polymorphic variants), while in two cases (15.4%) there were only *RET* polymorphisms (G691S variant alone or combined G619S/R982C variant). *RAS* mutations emerged in two *RET* wild-type cases and no mutations of either *RET* or *RAS* were detected in other two. Therefore, E-MTCs seem to be genetically heterogeneous, with a relative low prevalence of M918T somatic mutations (3 out of 7 *RET* mutated E-MTCs), but retain a more benign behavior regardless of the presence and type of underlying driver molecular alteration.

**Table 5:** *RET* and *RAS* genes mutational status in encapsulated MTC samples from previously published studies and present series.

Somatic mutations	Censi et al., 2019 (32)	Moura et al., 2021 (15)	Present series	Total cases
*RET* (%)	0/1 (0)	5/8 (62.5)	4/4 (100)	9/13 (69.2)
C620S	0	1	0	1
C630S	0	3	0	3
G691S or G691S/R982C	0	0	2	2
G691S/M918T/R982C	0	0	2	2
M918T	0	1	0	1
*HRAS* (%)	0/1 (0)	2/8 (25)	0/4 (0)	2/13 (15.4)
*RET+RAS wild type* (%)	1/1 (100)	1/8 (12.5)	0/4 (0)	2/13 (15.4)

In conclusion, the current research, along with previously published findings here reviewed, provides support for the idea that tumor encapsulation may represent a valid tissue biomarker of node-negative MTC, even in the setting of focal full-thickness capsular invasion, and thus be predictive of a better prognosis, similar to what is well established in follicular-derived thyroid carcinomas. We cannot say how much and in what way this can change the surgical approach to MTC, with the aim of limiting the number of unnecessary lymph node dissections for achieving disease remission with fewer side effects, as proposed by Machens and colleagues (18). From the literature review presented here, some E-MTCs with lymph node metastases have been described, although univocal definitions and shared criteria for the evaluation of tumor capsule, its continuity and integrity have not been provided.

Further studies would be also necessary to clarify the possible correlation of the presence of a complete capsule with other histological characteristics and with the molecular profile of the tumor, as well as larger longitudinal studies to better understand the outcome of patients with E-MTC on longer follow-up periods. Therefore, our proposal is to always describe the tumor capsule when present at the histopathological examination of a MTC, specifying its integrity and possible tumor invasion. Although this is not a necessary element for the diagnosis, as in other histotypes of thyroid cancer, this could be the first step in recognizing a standalone variant of MTC.

## Data Availability Statement

The raw data supporting the conclusions of this article will be made available by the authors, without undue reservation.

## Ethics Statement

The studies involving human participants were reviewed and approved by Milan Area 2 ethics committee. The patients/participants provided their written informed consent to participate in this study.

## Author Contributions

MA and AC designed the present study. AC conducted the literature research, collected clinical data and prepared the manuscript. MA, AD, UV, GC, EI, and AC performed clinical diagnosis and patient treatment and follow-up. MM, GL, and FP performed histopathological review of available samples at Fondazione IRCCS Ca’ Granda Ospedale Maggiore Policlinico, Milan, Italy. FE and GM were responsible for DNA analysis and *RET* and *RAS* mutations detection. MA, GM, and AD performed the critical revision of the manuscript. All authors read and approved the submitted version.

## Funding

This work was supported by Ricerca Corrente Funds from the Italian Ministry of Health to Fondazione IRCCS Ca’ Granda Ospedale Maggiore Policlinico.

## Conflict of Interest

The authors declare that the research was conducted in the absence of any commercial or financial relationships that could be construed as a potential conflict of interest.

## Publisher’s Note

All claims expressed in this article are solely those of the authors and do not necessarily represent those of their affiliated organizations, or those of the publisher, the editors and the reviewers. Any product that may be evaluated in this article, or claim that may be made by its manufacturer, is not guaranteed or endorsed by the publisher.
